# The American Medical Association

**Published:** 1879-06

**Authors:** 


### The American Medical Association.

In the city of Atlanta, Georgia, the thirtieth annual meeting of the American Medical Association was convened on the 6th of May, 1879, and, as usual, remained in session four days. On account of the distance from the homes of most of its members, it was scarcely expected that any considerable numbers would be present, but it was found that about three hundred and fifty delegates were in attendance from all parts of the country, the Southern States more particularly.

The *Boston Medical and Surgical Journal* describes the meeting in this wise:

" While the work in the sections, with but few exceptions, was less valuable than usual, and much time was wasted by reading papers unduly long and on unimportant subjects, the reports on the progress of the several departments of medical science, presented in general session by the chairman of the sections, were of more than usual interest and value, and were listened to with marked attention. The weather was extremely favorable for the Association, and indeed was almost too fair, as it had a tendency to lead the members to desert their work in the sections in the afternoon, in order to accept the hospitalities of private citizens, and to enjoy driving through the streets of this capital city, which has been termed the Chicago of the South, and which, in the combined advantages of beauty of location, healthfulness of climate, and cordiality of its citizens, is probably unexcelled by any city of its size in America.

The private entertainments were magnificent in their hospitality, and were characterized by such unmistakable appreciation and kindness as to be highly complimentary to the Association; they reflected the greatest credit upon the committee of arrangements and the profession and the citizens of Atlanta."

